# The Impact of a Theory-Based mHealth Intervention on Disease Knowledge, Self-efficacy, and Exercise Adherence Among Ankylosing Spondylitis Patients: Randomized Controlled Trial

**DOI:** 10.2196/38501

**Published:** 2022-10-20

**Authors:** Yuqing Song, Elizabeth Reifsnider, Yanling Chen, Ying Wang, Hong Chen

**Affiliations:** 1 West China School of Nursing West China Hospital Sichuan University Chengdu China; 2 Edson College of Nursing and Health Innovation Arizona State University Phoenix, AZ United States; 3 Department of Rheumatology and Immunology West China Hospital Sichuan University Chengdu China

**Keywords:** rheumatic disease, health belief model, mobile health, patient education, WeChat

## Abstract

**Background:**

Patient education is recommended as an integral part of disease management in ankylosing spondylitis (AS), a chronic rheumatic disease that predominantly affects young males and requires long-term disease management. Convenient and cost-effective approaches to deliver patient education are required to these patients.

**Objective:**

This study aimed to examine the effects of a theory-based educational intervention delivered through a social networking app, WeChat, on disease knowledge, self-efficacy, exercise adherence, and health outcomes in Chinese AS patients.

**Methods:**

This study was a single-blind randomized controlled trial conducted in a tertiary hospital in Chengdu, China. Eligible participants were randomly allocated to the intervention or control group. Participants in the control group received standard care. The intervention group received the health belief model (HBM)-based educational intervention, consisting of 4 individual educational sessions and educational information sharing through WeChat, the predominant social networking app in China. The primary outcomes were disease knowledge, self-efficacy, and exercise adherence. The secondary outcomes were disease activity and physical function. Data were collected at baseline and at the end of the intervention (12th week). Chi-square test, *t* test, Mann-Whitney *U* tests were used to examine the effects of educational intervention.

**Results:**

This study included 118 patients with AS. The majority of participants were male (93/118, 78.8%). Around half of them were married (56/118, 47.5%), never smoked (70/118, 59.3%), and had college educational level or above (62/118, 52.5%). At posttest, participants in the intervention group had higher disease knowledge (all *P*<.001) and self-efficacy (*P*<.001), and a larger proportion of participants in the intervention group adhered to regular exercise routines than those in the control group (*P*=.003). The within-group analyses for the intervention group showed increases in all scores of disease knowledge (all *P*<.001) and self-efficacy score (*P*<.001), but only correct answer score (*P*=.04) and general knowledge score (*P*=.002) of disease knowledge in the control group improved. The within-group analysis for the control group found a decline of physical function (*P*=.002) but no significant change in disease activity (*P*>.05). The within-group analysis for the intervention group showed no significant change in disease activity or physical function (*P*>.05). At posttest, no statistically significant difference was found on disease activity or physical function between the intervention and control groups (*P*>.05).

**Conclusions:**

The HBM-based educational intervention through WeChat can effectively improve patient disease knowledge, self-efficacy, and exercise adherence. WeChat is feasible and effective to deliver patient education for patients with chronic diseases such as AS. This mHealth intervention can be integrated into routine rheumatology care.

**Trial Registration:**

Chinese Clinical Trial Registry ChiCTR-IPR-16009293; https://tinyurl.com/swxt8xk7

## Introduction

### Background

Ankylosing spondylitis (AS) is a chronic rheumatic disease characterized by inflammatory back pain, morning stiffness, and reduced physical function that requires long-term management [1]. Regular disease management, including pharmacological therapy and exercise, is essential to control disease and prevent progression of the disease [1,2]. The inflammatory back pain and morning stiffness of AS patients improves with exercise but not with rest or inactivity [3]. Evidence reveals that exercise (such as stretching or aerobic exercise) can improve health outcomes, including pain, morning stiffness, and physical function, and the effects of exercise depend on patient adherence [4,5]. Exercise adherence refers to the extent to which people undertake the prescribed exercise from health care providers [5]. However, AS patients face severe challenges during disease management, such as lack of knowledge about the disease and nonadherence to medication and exercise [6-8]. Lack of knowledge about AS is a barrier to exercise and medication adherence [8]. Lack of exercise adherence negatively influences health outcomes among AS patients [8].

Patient education comprises educational activities designed to influence patient knowledge and health behaviors, enable patients to manage their disease, and optimize health outcomes [2,9]. Ndosi et al [10] revealed that patient education should aim at improving patient self-efficacy since self-efficacy is a predictor of health behaviors and health outcomes. Self-efficacy is defined as an individual’s confidence in performing a specific behavior [11]. Previous studies indicated that patient education can increase disease knowledge, self-efficacy, and adherence in arthritis patients [10,12,13]. However, only several published studies explored the effects of patient education among AS patients [14-20]. These interventions mostly reported small sample sizes, the lack of theoretical basis, and found limited effects on self-efficacy and adherence, and inconsistent results on health outcomes (eg, disease activity, physical function) [15-20]. The intervention delivery methods of many previous studies relied heavily on face-to-face interactions, which can be difficult due to travel restrictions, time constraints due to any number of factors, and costs of missing work [18,21].

Mobile health (mHealth) interventions can deliver timely health service and overcome the obstacles of time, distance, and cost [22]. The wide use of mobile phones has increased the possibility of delivering through health-focused interventions via apps [23]. In recent years, WeChat has been the most popular social networking app in China, with over 1 billion monthly active users [24]. WeChat can offer free message, voice/video calls, and enhance effective communication and information sharing [23,25]. WeChat has been used as a tool for educational interventions in patients with cancer, hypertension, coronary heart disease, and these studies reported positive effects [23,26-29]. Evidence revealed that AS patients need available education through phones and apps [30]. However, there is little evidence of an educational intervention through WeChat for AS patients.

AS patients may benefit from effective theory-based interventions to improve health behaviors and health outcomes (eg, disease activity, physical function). The health belief model (HBM) is developed to explain how to change health behaviors and focuses on an individual’s likelihood of engaging in healthy behaviors [11,31]. Previous studies revealed that the HBM is effective in developing interventions to change an individual’s beliefs and healthy behaviors in cancer, pulmonary tuberculosis patients [32,33]. However, this theory has not been used to develop an educational intervention for AS patients.

### Objectives

Based on these findings, a randomized controlled trial for AS patients was conducted and aimed to compare patient outcomes in a theory-based mHealth intervention via WeChat with standard care. Results regarding quality of life, depression, and selected clinical outcomes have been published elsewhere [34]. This paper describes the primary outcomes of this intervention, including disease knowledge, self-efficacy, exercise adherence, disease activity, and physical function. We hypothesized that the HBM-based mHealth intervention would improve the disease knowledge, self-efficacy, exercise adherence, physical function, and control disease activity of AS patients.

### Theoretical Framework

The key constructs of HBM consist of perceived susceptibility, perceived severity, perceived benefits, perceived barriers, cues to action and self-efficacy [11,35]. In HBM, health behaviors are based on people’s perceptions of susceptibility to and severity of health problems, barriers and benefits to enacting health behaviors and cues to action [31]. Self-efficacy can improve the efficacy of the model, and change subsequent health behaviors [11]. This intervention helped patients understand the severity of AS and provided strategies for managing their disease in order to improve self-efficacy, healthy behaviors, and achieve better health outcomes. We applied the theory to the AS intervention by using the constructs to guide the design of the intervention and match the intervention elements to the hypothesized outcomes. The primary goals of patient education are to transfer knowledge about disease and improve health behaviors [9]. Exercise adherence is a crucial health behavior related to AS patients, and self-efficacy is a predictor of health behaviors. This educational intervention was designed to explore short-term effects. Thus, we selected disease knowledge, self-efficacy, and exercise adherence as primary outcomes while health outcomes (ie, disease activity and physical function) were secondary outcomes.

## Methods

### Study Design

This study was a single-blinded randomized controlled trial conducted from March to December 2017. Written informed consents were obtained from all participants (legal guardians of participants under 18 years provided written informed consent) after they had received information about the study protocol.

### Sample Size Calculation

The sample size was determined by self-efficacy score based on our pilot trial, in which standard deviation was 1.47 and mean difference between the two groups was 0.86. With a power of 0.80 and α=.05 (2-sided), each group required 47 participants. The final sample size was 114, allowing a 20% dropout.

### Ethics Approval

The study was registered at the Chinese Clinical Trial Registry [ChiCTR-IPR-16009293], conducted in accordance with the Declaration of Helsinki, and approved by West China Hospital Medical Ethics Committee (ID: 20160364).

### Participants and Recruitment

The Department of Rheumatology and Immunology from a tertiary hospital in a large city, serving a population of Southwest China, was the site of recruitment. Potential participants were recruited via convenience sampling during their routine care. Participants were included if they (1) were diagnosed with AS according to the Modified New York Classification Criteria for AS, (2) were aged 14 years or older (participants under 18 years need written informed consent signed by legal guardians), (3) could speak and understand Chinese, (4) could use WeChat and had a WeChat account, and (5) were willing to participate in this randomized controlled trial study. We excluded participants if they (1) had severe cognitive or mental problems (comprehension or expression problems, using psychotropic drugs), (2) had other rheumatic diseases, or (3) were participating in other research programs.

### Randomization

To ensure participant assignment was truly randomized without human bias, a well-trained study coordinator generated a random allocation table in Excel (version 2010, IBM Corp). Before recruiting participants, the study coordinator placed randomized numbers in sealed opaque envelopes. Participants selected an envelope at random. Based on the selected number, participants were allocated to the intervention or control group. The researcher who collected the data and study coordinator who generated the random allocation table were blinded to group allocation.

### Intervention

#### Control Group

Patients in the control group received only standard care, including basic health advice appropriate for AS patients. Standard care was provided to the participants (including legal guardians of participants under 18 years) after they were recruited and completed the baseline assessment at the Department of Rheumatology and Immunology. Basic health advice was given in person by a nurse, and paper handouts were provided to the participants for their own education.

#### Intervention Group

The intervention group received the 12-week theory-based educational intervention delivered by WeChat plus standard care. The research team developed the educational intervention based on the HBM, a literature review of other social media–based interventions, expert consultation, and a pilot study. Finally, we identified the core content and corresponding HBM construct: basic knowledge of AS (perceived severity of disease, perceived susceptibility), medication (perceived benefit of preventive action), exercise (perceived benefit of preventive action), daily life management (perceived benefit of preventive action, cues to action), psychological support (perceived barriers to preventive action, supporting perceived self-efficacy), and self-assessment (perceived barriers to preventive action, cues to action; Table 1).

In the baseline assessment, we added participants as friends in WeChat and taught them how to use WeChat. The intervention consisted of 2 parts: online individual education sessions and educational information sharing. The first part included 4 individual educational sessions via WeChat video/voice calls on the 2nd, 4th, 8th, and 12th week. Each session was conducted for 20 to 30 minutes. Researchers contacted participants via telephone calls if they were absent from WeChat intervention sessions for 3 times. During the calls, research nurses built trusting relationships with participants, exploring their needs, problems managing disease, and psychological concerns. The nurses then used storytelling to illustrate potential severity. Nurse coaching during the calls helped the patients to establish cues to action to exercise, take medications, and promote health behaviors. The nurses used nurse coaching, verbal persuasion, and peer experience to address mood changes and support efforts toward self-efficacy. They taught participants how to assess their health conditions using validated instruments. They also encouraged self-efficacy by highlighting positive changes and helping patients manage self-doubt when lapses occurred. During the individual educational sessions, the nurses assessed participant knowledge about AS, problems, and health behaviors (eg, taking medication, exercising) related to AS, so the nurses could ensure whether the intervention positively influenced the target themes (eg, basic knowledge, medication) and provide targeted education. The second part consisted of selected pictures, videos, and articles on the WeChat public account about the core content of the intervention. The nurses sent links to online information once a week to participants. Moreover, participants could chat with the nurses at any time when they encountered problems with disease management.

**Table 1:** Content of the theory-based mobile health intervention.

Theme	Content	HBM^a^ construct	Method
Basic knowledge	Causes, pathogenesis, clinical symptoms, treatment, prognosis of AS^b^	Perceived severity of disease and its impact on future Perceived susceptibility to increasing limited mobility	One-to-one WeChat call: using storytelling to illustrate potential severity Linking to online information about AS basic knowledge
Medication	Treatment goals, importance of taking medication, medication management at home, side effect management, how to use a reminder for medication taking	Perceived benefits of preventive action to maintain current status	One-to-one WeChat call: using nurse coaching to highlight the benefit of medication taking Linking to online information about medication adherence
Exercise	Benefits of exercise, exercise type and intensity, helping reduce the obstacles to exercise, how to exercise at home	Perceived benefits of preventive action	One-to-one WeChat call: using nurse coaching to highlight the benefit of exercise regularly Online video Linking to online information on how to exercise
Daily life management	Physical posture, sleep instruction, diet, joint protection, quit smoking, etc	Perceived benefits of preventive action Creating cues to action	One-to-one WeChat call: using verbal persuasion and nurse coaching to highlight perceived benefits of healthy behaviors Linking to online information on daily life management
Psychological support	Psychological management strategies, providing patients with psychological support	Supporting perceived self-efficacy to manage disease Overcoming perceived barriers to preventive actions	One-to-one WeChat call: using nurse coaching, verbal persuasion, and peer experience to support efforts toward self-efficacy
Self-assessment	Teaching patients how to assess disease activity, function, and psychological status, etc	Creating cues to action Overcoming perceived barriers to preventive action	One-to-one WeChat call Sending online information on validated instruments

^a^HBM: health belief model.

^b^AS: ankylosing spondylitis.

### Measures

#### Demographic Information

Participant demographic data included age, gender, marital status, educational level, income, medical insurance, smoking status, and disease duration.

#### Primary Outcomes

Primary outcomes included disease knowledge, self-efficacy, and exercise adherence. Disease knowledge refers to the level of knowledge about AS in patients with AS [36]. In this study, patients’ level of knowledge of AS was assessed by the Assessment of Knowledge in Ankylosing Spondylitis Patients [37]. This instrument is divided into 4 areas: (1) general knowledge, etiology, symptoms, blood tests; (2) B27 antigen and inheritance; (3) drug treatment and physical therapy; and (4) joint protection, pacing, and priorities. The instrument has 14 questions with 72 potential answers, but only 25 answers are correct. The correct answer score (maximum possible=25) is obtained by giving 1 point to each correct answer, and the correct item score (maximum possible=14) is obtained by giving 1 point to each question with all the correct answers [36]. Higher scores indicate higher levels of knowledge about AS. Cronbach alpha of this instrument was .729 in this study.

Self-efficacy is defined as an individual’s confidence in performing a specific behavior [11]. In this study, self-efficacy was measured using the Arthritis Self-Efficacy Scale–8 (ASES-8) [38]. ASES-8 included 2 items for pain subscales, 4 items from other symptoms subscales, and 2 items that related to keeping pain and fatigue from interfering with things the patients want to do [39]. The final score is 0 to 10, with higher scores indicating higher self-efficacy. ASES-8 had good reliability, validity, and adaptability in arthritis patients [38,40]. Cronbach alpha of ASES-8 in this study is .913.

Exercise adherence refers to the extent to which people undertake the exercise prescribed by health care providers [5]. In this study, adherence to exercise was examined using a self-reported statement as used in previous studies [16,41]: frequency of exercise per week (0, occasionally, 1-2 days, 3-4 days, 5-6 days, daily).

#### Secondary Outcomes

Disease activity and physical function reflect the main aspects of health outcomes among AS patients. Disease activity was measured by the Bath Ankylosing Spondylitis Disease Activity Index (BASDAI) [42], a patient-reported scale to assess the severity of major symptoms (fatigue, spinal pain, joint pain and swelling, areas of localized tenderness, and morning stiffness) in AS patients. Physical function was measured by the Bath Ankylosing Spondylitis Functional Index (BASFI) [43], a patient-reported scale to assess patient function (eg, bending, reaching, changing position) and the ability to cope with everyday life. The final BASDAI and BASFI scores ranged from 0 to 10, with higher scores indicating higher disease activity and worse physical function; Cronbach α=.740 (BASDAI) and α=.956 (BASFI).

### Data Collection

Data were collected at baseline and the 12th week. If participants had difficulty in reading or writing, the researcher would help them complete questionnaires. Baseline data were collected from participants and medical records. The posttest data were collected from participants when they came to the rheumatology clinic for routine care or through an online survey platform [44] or through telephone/WeChat call.

### Statistical Analyses

Data were analyzed using SPSS (version 22.0, IBM Corp) software. An intention-to-treat principle was used for analyses, and the last observation carried forward method was used for missing data assessment. Data were described as mean and standard deviation, median and interquartile range, and frequency and percentage. Independent sample *t* test, Mann-Whitney *U* test, and chi-square test were used to compare data between the intervention and control groups. Paired sample *t* tests were used to analyze the changes in outcomes from baseline to the 12th week within each group in continuous variables. *P*<.05 was considered statistically significant.

## Results

### Participants

**Figure 1:**
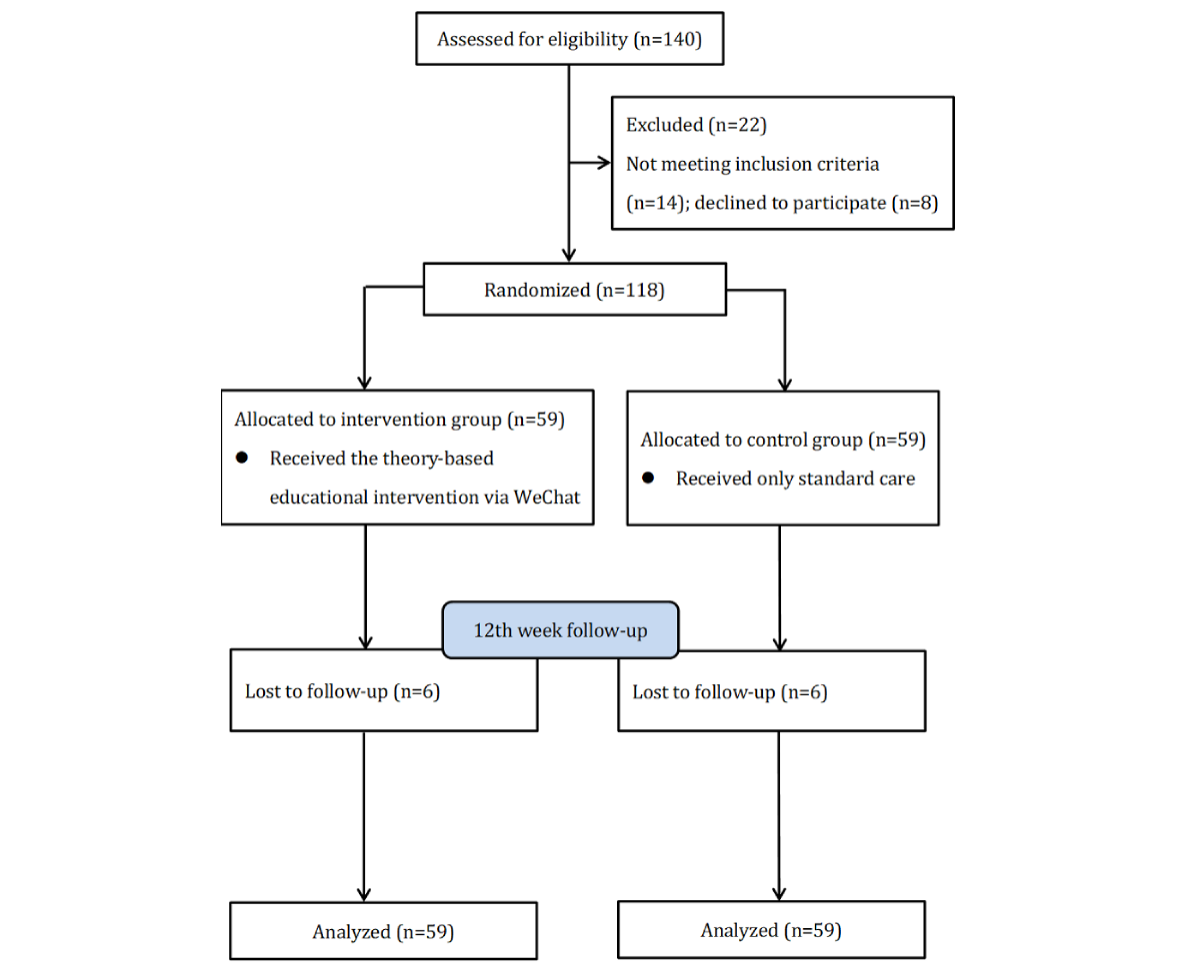
Flow diagram of the study.

A total of 118 participants were included and randomly allocated into the intervention (n=59) or control group (n=59). A total of 89.8% (106/118) of participants completed the study. Additionally, we included 118 participants in data analyses because the intention-to-treat principle was used. Figure 1 shows the study flowchart.

### Baseline Characteristics of Participants

The average age of participants was 29.9 years. The majority of participants were male (93/118, 78.8%). Around half of participants were married (56/118, 47.5%), never smoked (70/118, 59.3%), and had college educational level or above (62/118, 52.5%). There was no statistically significant difference in the variables between the 2 groups (Table 2).

**Table 2:** Baseline characteristics of participants in the intervention and control group.

Characteristic			Total (n=118)		Intervention (n=59)		Control (n=59)		*t*^a^/χ^2b^/Z^c^		*P* value
Age (years), mean (SD)			29.9 (8.23)		30.8 (8.82)		29.1 (7.58)		1.142^a^		.26
**Gender, n (%)**			—^d^		—		—		0.457^b^		.50
	Male	93 (78.8)		45 (76.3)		48 (81.4)		—		—	
	Female	25 (21.2)		14 (23.7)		11 (18.6)		—		—	
**Educational level, n (%)**			—		—		—		0.218^b^		.90
	Junior high school or below	30 (25.4)		15 (25.4)		15 (25.4)		—		—	
	Senior high school	26 (22.0)		12 (20.3)		14 (23.7)		—		—	
	College or above	62 (52.5)		32 (54.2)		30 (50.8)		—		—	
**Marital status, n (%)**			—		—		—		0^b^		>.99
	Single/divorced	62 (52.5)		31 (52.5)		31 (52.5)		—		—	
	Married	56 (47.5)		28 (47.5)		28 (47.5)		—		—	
**Monthly per capita income (￥), n (%)**			—		—		—		1.720^b^		.63
	<2200	34 (28.8)		16 (27.1)		18 (30.5)		—		—	
	2200-3300	33 (28.0)		15 (25.4)		18 (30.5)		—		—	
	3300-5500	23 (19.5)		11 (18.6)		12 (20.3)		—		—	
	>5500	28 (23.7)		17 (28.8)		11 (18.6)		—		—	
**Medical insurance, n (%)**			—		—		—		0.160^b^		.69
	Self-pay	82 (69.5)		40 (67.8)		42 (71.2)		—		—	
	Medical insurance	36 (30.5)		19 (32.2)		17 (28.8)		—		—	
**Smoking status, n (%)**			—		—		—		1.502^b^		.47
	Current smoking	36 (30.5)		19 (32.2)		17 (28.8)		—		—	
	Never smoking	70 (59.3)		36 (61.0)		34 (57.6)		—		—	
	Quit smoking	12 (10.2)		4 (6.8)		8 (13.6)		—		—	
Symptom duration, median (IQR)			5.00 (6.25)		6.00 (7.00)		5.00 (7.00)		–0.346^c^		.73
Diagnosis duration, median (IQR)			3.00 (6.00)		3.00 (6.00)		3.00 (6.00)		–0.577^c^		.56
**Knowledge of AS^e^**											
	Correct item score, mean (SD)	6.41 (3.08)		6.64 (2.92)		6.17 (3.24)		0.836^a^		.41	
	Correct answer score, mean (SD)	15.29 (4.91)		15.61 (4.58)		14.97 (5.24)		0.711^a^		.48	
	General knowledge, mean (SD)	5.21 (1.86)		5.19 (1.82)		5.24 (1.91)		–0.148^a^		.88	
	B27 antigen and inheritance, median (IQR)	1.00 (1.00)		1.00 (1.00)		1.00 (1.00)		–0.938^c^		.35	
	Drug treatment and physical therapy, mean (SD)	6.05 (2.16)		6.34 (1.91)		5.76 (2.37)		1.456^a^		.15	
	Joint protection, pacing and priorities, mean (SD)	3.22 (1.35)		3.34 (1.17)		3.10 (1.52)		0.952^a^		.34	
Self-efficacy, mean (SD)			6.29 (1.93)		6.40 (1.91)		6.18 (1.97)		0.624^a^		.53
Disease activity, mean (SD)			3.28 (1.89)		3.15 (1.81)		3.41 (1.97)		–0.769^a^		.44
Physical function, median (IQR)			0.60 (1.70)		0.60 (1.90)		0.60 (1.50)		–0.586^c^		.56
**Adherence to exercise, per week** **, n (%)**			—		—		—		10.450^b^		.06
	0	9 (7.6)		1 (1.7)		8 (13.6)		—		—	
	Occasionally	66 (55.9)		34 (57.6)		32 (54.2)		—		—	
	1 or 2 days	14 (11.9)		9 (15.3)		5 (8.5)		—		—	
	3 or 4 days	11 (9.3)		8 (13.6)		3 (5.1)		—		—	
	5 or 6 days	1 (0.8)		0 (0)		1 (1.7)		—		—	
	Daily	17 (14.4)		7 (11.9)		10 (16.9)		—		—	

^a^independent sample *t* test.

^b^chi-square test.

^c^Mann-Whitney *U* test.

^d^Not applicable.

^e^AS: ankylosing spondylitis.

### Primary Outcomes

The correct item score, correct answer score, and 4 area scores of knowledge of AS and self-efficacy scores in the intervention group were significantly higher than the control group after the intervention (all *P*<.001). A larger proportion of participants in the intervention group adhered to regular exercise after the intervention compared with the control group (*P*=.003, Table 3). The within-group analyses for the intervention group showed significant increases in all scores of AS knowledge and self-efficacy scores (all *P*<.001). The within-group analyses for the control group detected increases in correct answer score (*P*=.04) and general knowledge score (*P*=.002), but no significant difference in self-efficacy score, other scores of knowledge of AS including the correct item score (all *P*>.05, Table 4).

**Table 3:** Comparison of outcomes between groups at posttest.

Characteristic		Intervention (n=59)	Control (n=59)		*t*^a^/Z^b^/χ^2c^		*P* value
**Knowledge of AS^d^**							
	Correct item score, mean (SD)	11.81 (2.44)	6.83 (3.34)	9.249^a^		<.001^e^	
	Correct answer score, mean (SD)	22.49 (3.35)	16.05 (5.17)	8.022^a^		<.001^e^	
	General knowledge, mean (SD)	7.20 (1.31)	5.80 (1.75)	4.943^a^		<.001^e^	
	B27 antigen and inheritance, median (IQR)	2.00 (1.00)	1.00 (1.00)	–7.139^b^		<.001^e^	
	Drug treatment and physical therapy, mean (SD)	8.48 (1.01)	5.95 (2.31)	7.706^a^		<.001^e^	
	Joint protection, pacing and priorities, mean (SD)	4.53 (0.80)	3.39 (1.29)	5.766^a^		<.001^e^	
Self-efficacy, mean (SD)		7.60 (1.50)	6.41 (2.04)		3.612^a^		<.001^e^
Disease activity, mean (SD)		2.95 (1.71)	3.41 (1.76)		–1.434^a^		.15
Physical function, median (IQR)		1.00 (1.40)	1.40 (1.60)		–1.764^b^		.08
**Adherence to exercise, per week, n (%)**		—^f^	—		18.028^c^		.003^e^
	0	1 (1.7)	6 (10.2)	—		—	
	Occasionally	11 (18.6)	28 (47.5)	—		—	
	1 or 2 days	15 (25.4)	8 (13.6)	—		—	
	3 or 4 days	12 (20.3)	7 (11.9)	—		—	
	5 or 6 days	6 (10.2)	4 (6.8)	—		—	
	Daily	14 (23.7)	6 (10.2)	—		—	

^a^independent sample *t* test.

^b^Mann-Whitney *U* test.

^c^chi-square test.

^d^AS: ankylosing spondylitis.

^e^*P*<.01.

^f^Not applicable.

**Table 4:** Comparison of outcomes within the intervention and control groups.

Characteristic and group				Pretest, mean (SD)		Posttest, mean (SD)		Difference of means (95% CI)		*t* ^a^		*P* value
**Knowledge of AS^b^**												
	**Correct item score**											
		IG^c^	6.64 (2.92)		11.81 (2.44)		5.17 (4.22 to 6.11)		10.953		<.001^d^	
		CG^e^	6.17 (3.24)		6.83 (3.34)		0.66 (–0.12 to 1.44)		1.694		.10	
	**Correct answer score**											
		IG	15.61 (4.58)		22.49 (3.35)		6.88 (5.55 to 8.21)		10.345		<.001^d^	
		CG	14.97 (5.24)		16.05 (5.17)		1.08 (0.06 to 2.11)		2.126		.04^f^	
	**General knowledge**											
		IG	5.19 (1.82)		7.20 (1.31)		2.02 (1.52 to 2.51)		8.123		<.001^d^	
		CG	5.23 (1.91)		5.80 (1.75)		0.56 (0.21 to 0.91)		3.231		.002^d^	
	**B27 antigen and inheritance**											
		IG	0.75 (0.76)		2.29 (0.85)		1.54 (1.28 to 1.80)		11.988		<.001^d^	
		CG	0.86 (0.75)		0.92 (0.75)		0.05 (–0.17 to 0.27)		0.465		.64	
	**Drug treatment and physical therapy**											
		IG	6.34 (1.91)		8.47 (1.01)		2.14 (1.59 to 2.68)		7.794		<.001^d^	
		CG	5.76 (2.37)		5.95 (2.31)		0.19 (–0.22 to 0.59)		0.919		.36	
	**Joint protection, pacing, and priorities**											
		IG	3.34 (1.17)		4.53 (0.80)		1.19 (0.84 to 1.53)		6.840		<.001^d^	
		CG	3.10 (1.52)		3.39 (1.29)		0.29 (–0.12 to 0.70)		1.404		.17	
**Self-efficacy**												
		IG	6.41 (1.91)		7.60 (1.50)		1.19 (0.72 to 1.66)		5.055		<.001^d^	
		CG	6.18 (1.97)		6.41 (2.04)		0.22 (–0.16 to 0.62)		1.178		.24	
**Disease activity**												
		IG	3.15 (1.81)		2.96 (1.71)		–0.19 (–0.70 to 0.32)		–0.754		.45	
		CG	3.41 (1.97)		3.41 (1.76)		0 (–0.52 to 0.52)		–0.005		>.99	
**Physical function**												
		IG	1.61 (2.23)		1.67 (1.79)		0.07 (–0.44 to 0.57)		0.269		.79	
		CG	1.25 (1.63)		1.91 (1.65)		0.67 (0.26 to 1.07)		3.320		.002^d^	

^a^paired sample *t* test.

^b^AS: ankylosing spondylitis.

^c^IG: intervention group.

^d^*P*<.01.

^e^CG: control group.

^f^*P*<.05.

### Secondary Outcomes

At posttest, there was no difference in disease activity or physical function between the intervention and control groups (*P*>.05). The within-group analyses for the intervention group showed no significant change in disease activity or physical function (*P*>.05). The within-group analyses for the control group detected a decline in physical function (*P*=.002), but no significant change in disease activity (*P*>.05, Table 3 and Table 4).

## Discussion

### Principal Findings

This study explored the effects of the theory-based educational intervention through WeChat among Chinese patients with AS. Our findings demonstrated that this intervention was feasible and beneficial for improving patient disease knowledge, self-efficacy, and exercise adherence, which was in line with previous studies [12,16,41]. The educational intervention delivered by WeChat can increase access to health care providers for participants, teach knowledge and skill of disease management, and have positive effects in AS patients.

We found that the theory-based educational intervention can increase patient knowledge about AS, which was in line with prior studies [16,41], and corresponds with educating the patients on perceived severity of the disease and perceived susceptibility to increased limited mobility without action. Haglund et al [30] revealed that 43% of spondyloarthritis patients had educational needs. Moreover, patient knowledge levels of AS in this study were relatively low compared with previous studies [36]. In our study, the research nurses provided knowledge of managing AS, which may increase patient knowledge levels of AS.

The educational intervention via WeChat can effectively improve self-efficacy of AS patients, a finding similar to prior studies [18,45]. Self-efficacy can be enhanced through direct experience, alternative experience, and verbal persuasion [46]. In this study, participants gained knowledge and peer experience about disease management through educational information and nurse coaching by praising small accomplishments, which increased their perceived self-efficacy. Learning about useful experiences of others can inspire patients to try strategies to manage disease [47]. Seeing the adaptations of others to AS helps patients manage their disease better and improves confidence in coping with disease, which increases their self-efficacy to manage the disease. Persuasion from research nurses can help patients successfully manage their conditions [48]. That these skills may enhance patient confidence in managing disease and improving their self-efficacy was shown in our intervention.

This intervention effectively improved patient self-efficacy which, in turn, may have contributed to higher adherence to exercise. In this study, a larger proportion of participants in the intervention group adhered to regular exercise compared with the control group after the intervention. The finding was in line with earlier studies [16,41]. Self-efficacy is an important factor influencing exercise behavior in AS patients [49]. Our intervention helped patients perceive the severe consequences of AS, educated them on the importance of disease management, and taught them skills to manage their condition, which may have prompted regular exercise and helped them develop cues to action in their daily lives. In addition, the intervention delivered through WeChat may make it easier to exercise at home. These issues may enhance patient exercise adherence.

The results of this study did not detect significant differences in disease activity and physical function except for a decline in physical function in the control group. Previous reports on the efficacy of patient education on disease activity and physical function are inconsistent [14-18,20]. In our study, patients had relatively low disease activity and functional limitation, and these variables may be difficult to modify. Our 12-week intervention period may not be long enough to detect significant changes in biomarkers, such as disease activity, function, etc. Educational intervention may not produce a direct effect on health outcomes [2]. Thus, future studies should explore the long-term effects of educational intervention on health outcomes.

### Limitations

This study had several strengths. An assessor blinded to group assignment collected pretest and posttest data to reduce biased responses. Furthermore, using HBM might increase the efficacy of this intervention. Finally, we used an intention-to-treat analysis with multiple imputations for missing data to reduce bias in assessment of treatment effects.

This study had several potential limitations. First, we only recruited patients from a tertiary hospital who were able to use WeChat. Although the use of smartphone and internet access are relatively ubiquitous, the use of WeChat limits the generalization of findings to all Chinese AS patients. Second, patient views and cost-effectiveness analysis are important to evaluate and improve this educational intervention, but we did not collect these data because of limited time and financial support. Third, we collected outcomes at 12 weeks, but the effects of WeChat-based education on health outcomes may only become apparent in a long-term.

### Conclusions

We demonstrated that the theory-based educational intervention delivered through WeChat, led by experienced nurses, was feasible and effective to improve AS patient disease knowledge, self-efficacy, and exercise adherence in a short-term. WeChat can deliver timely health service for patients with no available time or living in rural communities. During the COVID-19 pandemic period, the intervention approach may help health care providers provide continuous rheumatology care. We suggest that this intervention can be integrated into routine rheumatology care. Future studies should explore long-term effects of this intervention.

## Appendix

Multimedia Appendix 1 CONSORT-eHEALTH checklist (V 1.6.1).

## Abbreviations

BASDAI: Bath Ankylosing Spondylitis Disease Activity Index
BASFI: Bath Ankylosing Spondylitis Functional Index
AS: ankylosing spondylitis
ASES-8: Arthritis Self-Efficacy Scale–8
HBM: health belief model
mHealth: mobile health
